# Medical Student Learning Experience With Attending or Resident Preceptors in the Emergency Department

**DOI:** 10.7759/cureus.47285

**Published:** 2023-10-18

**Authors:** Collyn Murray, Cody Stauffer-Macdowell, Christina Shenvi

**Affiliations:** 1 Emergency Medicine, University of North Carolina at Chapel Hill School of Medicine, Chapel Hill, USA; 2 Emergency Medicine, Mease Dunedin Hospital, Dunedin, USA

**Keywords:** medical student education, emergency medicine precepting, emergency medicine rotation, fourth-year medical student education, emergency medicine training

## Abstract

Objectives

The emergency department (ED) provides a unique learning environment for medical students. However, environment, patient, and preceptor factors limit standardized teaching. We explored the most effective educational interactions for fourth-year medical students during an emergency medicine (EM) clerkship designed to allow clinical interaction with both residents and faculty.

Methods

This is an exploratory, prospective, needs assessment study of objective cards and surveys submitted by medical students as part of their month-long fourth-year clinical rotation at a tertiary care academic ED. Students marked which topics or procedures they had reviewed, and who had precepted them. In an exit survey, students were asked to rate how often they received individualized teaching and whether their educational goals were met when working with residents and attendings. Qualitative and quantitative data were collected anonymously with institutional review board (IRB) exemption.

Results

Shift card data was collected from 69 of the rotating students. Attendings tended to precept visual diagnostics while residents tended to teach technical procedures. Forty-four students completed the exit survey. Results showed that students felt they received individualized teaching from both attendings and residents (7.9 and 8.0 respectively, p = 0.059). Students felt their goals were met more when reporting to the residents than the attendings but not significantly so (8.6 and 8.0, respectively, p = 0.088). Additional themes were that students wanted more individualized experiences with the attendings and requested more dedicated teaching shifts.

Conclusions

Fourth-year medical students in the ED felt they received individualized teaching on most shifts. They reported their education goals were met as often when working with residents as with attendings; however, interactions feature different educational content. Clerkship curricula design would benefit from resident and attending-directed teaching experiences to optimize the educational experience in the ED.

## Introduction

Education of medical students in the emergency department (ED) is often led by either residents or attending physicians. The individual teaching the student may vary based on interest, skills, level of engagement, and how busy the clinical department is. However, the quality of teaching that students receive when precepted by attendings compared to residents has not been previously assessed. Acting internships in emergency medicine (EM) are a popular fourth-year medical school rotation among students intending to apply to EM as well as those applying in other fields. The ED provides a unique learning opportunity for students to understand the approach to and workup of the undifferentiated patient, acute resuscitation, and the role of the ED in the healthcare system. One of the most important contributors to the medical student experience on any rotation is the person to whom they directly report. This individual will be the one to provide feedback, on-shift education, guidance, and cognitive apprenticeship. In 2015, ACEP released revised guidelines for undergraduate education in EDs. The goal of the rotation should be to teach students the principles of emergency medicine, which include focused history taking, focused examinations, teamwork, diagnostic reasoning, and critical thinking [1]. A 2006 task force report recommended that fourth-year EM curricula include objective-based learning, core content, feedback/evaluation, and flexible implementation [2]. However, these do not give a set method to teach these skills. There is no current consensus or best practices for the optimal way to implement an EM rotation or for how students should integrate into the ED workflow. In different EDs, medical students may present and report directly to attending physicians, residents, or some combination of the two [2,3]. This allows flexibility and individualization at different programs but is also an opportunity to study and identify best practices and the best learning environment for medical student education.

During their clinical rotation, medical students participated in residency and individual didactics, simulation, and clinical shifts. Students were assessed through faculty and resident evaluations, completion of an objective-based topics and procedure card (described below), and an end-of-rotation exam. Clinical shifts were eight hours scheduled in the same format as residents and attendings. Students rotated in both the main ED and the minor treatment area, and on teaching shifts. During their main ED shifts, a medical student was precepted by a senior resident (PGY 2 or 3) or an attending depending on the bay location of their scheduled shift. In the minor treatment area, medical students reported to attendings only. Teaching shifts were composed of a senior resident and two to three of the rotating medical students. During this shift, residents provided clinical teaching in the department regarding patient care or equipment and staffed patients primarily evaluated by medical students in a junior attending role prior to staffing with the primary attending. Students worked with a variety of residents and attendings during their clinical rotations.

Our goal was to examine medical student education in the ED to determine where and with whom the fourth-year medical students felt their educational objectives were being best met. Specifically, we studied their experience reporting directly to attending physicians compared to reporting to senior residents.

## Materials and methods

This was an exploratory, prospective needs assessment study of fourth-year medical students during a month-long acting internship rotation. Data were collected during a 10-month period at a single tertiary care academic ED. At the start of each month-long rotation, all students were given a procedure/learning card on which to mark which topics they had discussed with an attending or resident, and which procedures they had performed. The items listed on the card included common procedures that could be performed by medical students and a predetermined set of the most important topics or patient presentations that students should see or discuss in the ED. The purpose of creating the card was to help prompt students to ensure they encounter those clinical entities and encourage them to participate in the procedures. During the course of the rotation, the students would add content to the cards after each shift. Examples of discussion topics included headache, altered mental status, and chest pain, among others. The procedures listed included peripheral intravenous access, electrocardiogram (ECG) interpretation, laceration repair, and lumbar punctures, among others. Prior exposure to or experience with all of these procedures would vary based on the student's previous rotations and clinical experiences.

At the end of the rotation, students submitted their completed procedure/learning card. In addition, the students completed an exit survey to rate how often they received individualized teaching and whether their education goals were met when working with residents or attendings on a 10-point Likert scale.

Data were analyzed using Microsoft Excel (Microsoft Corporation, Redmond, WA). Descriptive statistics, including mean scores were calculated and differences were compared using paired T-tests to determine statistical significance. Medical students were also asked to provide open-ended feedback and potential improvements at the conclusion of the survey in the form of a free text box. Qualitative and quantitative data were collected anonymously, and the study was granted IRB exemption.

## Results

Shift card data was collected from 69 rotating students. Given the structure of the rotation, most medical student shifts were precepted by attendings, and discussions of major topics were most often led by the attending. Potential preceptors for procedures included attendings, residents, and nursing staff. Attendings more often precepted visual diagnostics, including ECG (61%) and CXR (65%) interpretation while residents more often provided direct teaching of technical procedures including central venous access (64%) and focused assessment with sonography in trauma (FAST) exams (65%) with attending supervision. Nursing staff also contributed to medical student teaching as primary preceptors for phlebotomy, peripheral intravenous access, and placement of patients on the cardiac monitor.

**Figure 1:**
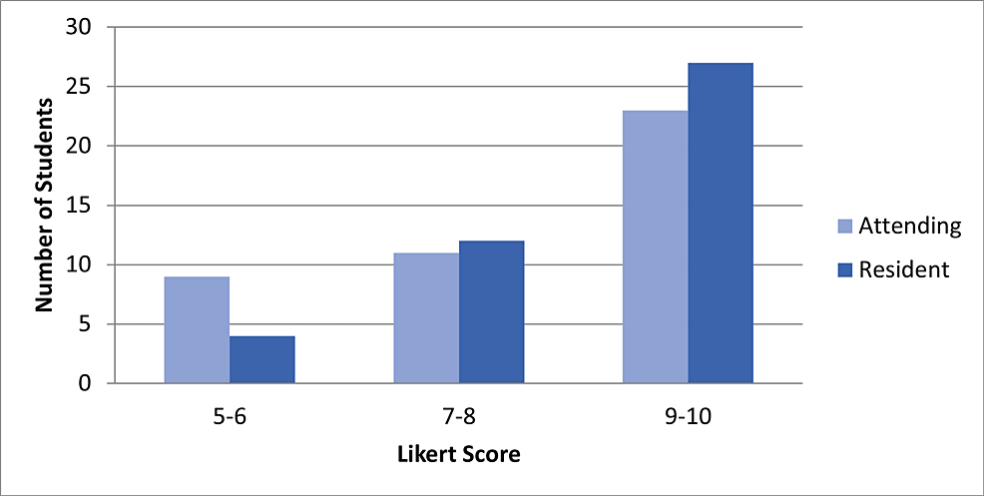
Rating of learning goal achievement by students with attendings or residents

**Figure 2:**
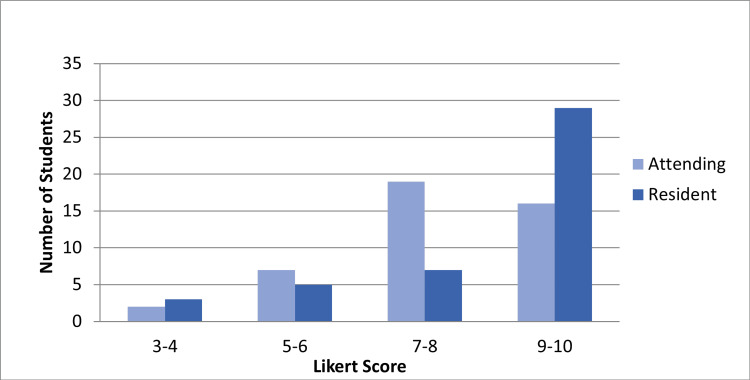
Rating of individualized teaching by students with attendings and residents

**Figure 3:**
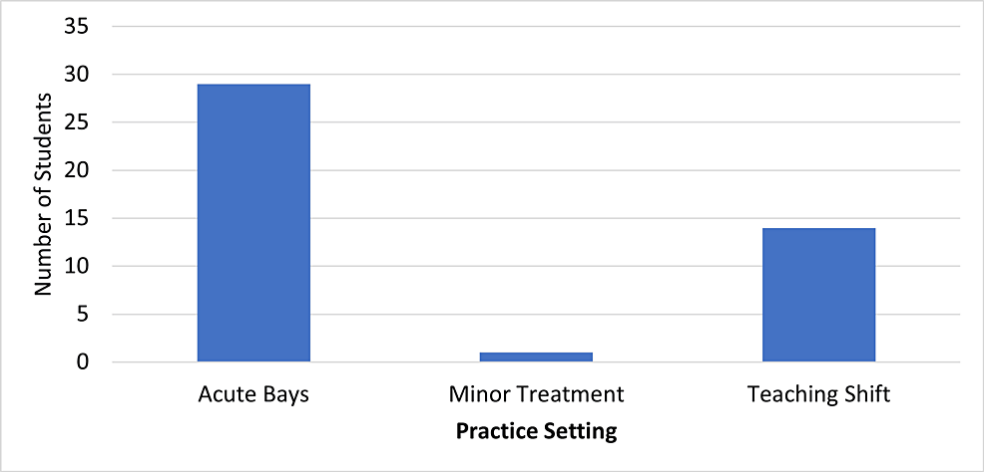
Ranking of practice setting in meeting educational goals

Forty-four students completed the exit survey (64%). Students felt their goals were met more when reporting to the residents than attending, but this difference was not statistically significant (mean = 8.6 and 8.0, respectively, p=0.088) (Figure 1). With regards to the educational experience, students felt they received individualized teaching (Figure 2) more from residents than attendings (mean = 8.5 and 7.9, p=0.058). In terms of practice setting (Figure 3), students preferred the higher acuity areas over teaching shifts and lower acuity areas (65.9%, 31.8%, and 2.3%). The most frequent recommendations from the open-ended portion were that students wanted more individualized experiences with attendings, more dedicated teaching shifts, and more simulation experience.

## Discussion

Clinical acting internships in the ED provide an excellent learning opportunity for fourth-year medical students. The rotation offers the opportunity to diagnose a wide variety of medical conditions as well as exposure to various medical specialties. As EM becomes more popular across the country, more medical schools are offering it as a rotation for both third and fourth-year students [4,5]. Little is known about the most effective way to set up a clinical rotation. Many authors have examined teaching in the ED and have offered guidelines similar to those developed by the American College of Emergency Physicians (ACEP) but have not come up with definitive best practices [2,6-8].

The same factors that make the ED an ideal learning environment for acute care can also present challenges to effective medical student education. The fast pace and acute nature of the patients can limit the amount of teaching for students. Our results show that students reported excellent learning when precepted by both attendings and residents. It further indicated that residents and attendings provide directed teaching on different but complementary topics. We believe that residents are valuable medical student supervisors and complement attending instruction in a busy department. In addition, their inclusion provides a robust opportunity for the resident to develop their own skills as an educator and a preceptor. Prior studies in surgical rotations have demonstrated that students feel comfortable being educated primarily by residents [9]. Residents are closer in skill level and age to students and may have an easier time explaining ideas and understanding the student’s perspective and knowledge gaps. A recent study by Byrne et al. showed similar results when comparing student perceptions of residents as educators in the ED [10], with similar results in simulation [11].

We have compared two different teaching paradigms for medical students: attending- and resident-driven. The results of our study have shown roughly equivalent learning opportunities and achievement of learning objectives for students who were precepted by both faculty and residents. This supports the growing evidence that residents play a key role in the education of medical students. Since students received excellent education when reporting to either the attending or the senior resident, institutions may have at their disposal a more robust teaching cohort than previously thought. The latter may allow for increased flexibility in the teaching structure and better accommodate the teaching needs in different locations in the ED based on patient acuity, required workflow, and patient needs on any given day. For example, students could be taught by residents when the pace in the ED is slower and by attendings when the pace limits the amount of time residents can spend on teaching. Resident preparation for this role, particularly with instruction in technical procedural skills, should be considered in clerkship design. A mixed model of student education, one in which residents and attendings are available as educators, would also allow residents the opportunity to develop their teaching skills as a part of the Liaison Committee on Medical Education (LCME) and Accreditation Council for Graduate Medical Education requirements [12,13].

The medical students in our study also indicated they would like to spend more time in the acute bays and on teaching shifts. This desire is likely because patients with more complex needs and presentations afford a greater opportunity for learning from the patients and receiving teaching from the residents or attendings. They felt their learning objectives were met more often in these scenarios versus minor care areas. As the acute areas typically house complex patients where time is critical, a mixed model would give students not only a variety of learning experiences but also more opportunities for direct teaching while maintaining a realistic experience as an emergency medicine physician.

Since the coronavirus disease 2019 (COVID-19) pandemic and with growing awareness of the high rates of burnout among Emergency Physicians [14], the Match in EM in 2023 had the highest number of unfilled positions in the specialty's history [15]. The reasons for the catastrophic drop in the match rate are multifactorial, including a growth in the number of available positions, and a decrease in the number of applicants. The latter factor played the greatest role. This drop in applications makes it all the more important that programs provide an outstanding educational experience for medical students in the ED. By improving the education and experience of medical students, it may be possible to attract more applicants to the field, thus curbing the negative ongoing trends. Other systemic changes, such as reducing the burden on boarding patients, providing adequate nursing and physician staffing, and ensuring there is time for teaching on shift, are other important factors that impact the educational experience of learners in the ED. 

Limitations

Our study is limited because it is a relatively small, single-center study. A larger sample size is needed across multiple clinical environments in which residents and attendings are preceptors to better characterize learning goal achievement. Other departments may have different workflows or patient care needs that could limit their flexibility or capacity for medical student education and variation in clerkship design. Further, patient care requirements and staffing may limit resident involvement in direct clinical teaching, which would impact a student’s ability to meet their learning goals. Like Byrne et al., in precepting situations where both residents and attendings are available, students may approach each preceptor with different goals in mind, which may explain the similarities in experience. A more robust qualitative evaluation would help provide insight into the same. Finally, this study relied on learner-centered feedback and assessment of the quality of the teaching. A more objective measure of learning outcomes, such as performance in the clinical setting or on assessments, would provide more robust data regarding the effectiveness of the varied instructions. Input from both residents and attendings regarding their teaching experiences is also needed to further explore the benefits of a mixed model of education.

## Conclusions

Our data support that medical students frequently receive individualized teaching and meet their learning objectives when working directly with either an attending or a senior resident. This supports a model for acting internship rotations in which students report to either, as the clinical environment allows, and should be considered along with objective-based learning and feedback as a key consideration in EM clerkship design. Providing a robust and meaningful learning experience for students is important to prepare them for residency. In addition, given recent trends in residency applications, a positive learning experience and environment may encourage more students to apply to the field of emergency medicine.
